# Advancing Toward the UNAIDS 95-95-95 Targets in Sierra Leone: A Narrative Review of Progress, Persistent Gaps, and Policy Priorities

**DOI:** 10.5334/aogh.5152

**Published:** 2026-03-26

**Authors:** Augustus Osborne, Ibrahim Franklyn Kamara, Sulaiman Lakoh, Mariama Mustapha, Alren Vandy, Morie Vandi, Sia Morenike Tengbe, Osman Sankoh

**Affiliations:** 1 Institute for Development, Western Area, Freetown, Sierra Leone; 2 Family and Reproductive Health Unit, Health Systems and Services Cluster, World Health Organization Country Office-Sierra Leone; 3 Centre of Tropical Medicine and Travel Medicine, Amsterdam UMC, Department of Infectious Diseases, University of Amsterdam, Amsterdam, The Netherlands; 4 Department of Infectious Diseases, Division of Internal Medicine, Amsterdam UMC, Netherlands; 5 Ministry of Health, College of Medicine and Allied Health Sciences, University of Sierra Leone, Sierra Leone; 6 College of Medicine and Allied Health Sciences, New England Ville, Freetown, Sierra Leone; 7 KYM Consultancy, Wilkinson Riad, Freetown, Sierra Leone; 8 World Health Organization Country Office, 21A-B Riverside Drive, Off Kingharman Road, Freetown, Sierra Leone; 9 Ministry of Health, 4^th^ Floor, Youyi Building, Freetown, Sierra Leone; 10 Centre for Health Research and Training, University of Management and Technology (UNIMTECH), Kissy Dockyard, Freetown, Sierra Leone; 11 College of Medical Sciences, Bo Campus, Njala University, Bo, Sierra Leone; 12 School of Public Health, Faculty of Health Sciences, University of the Witwatersrand, Johannesburg, South Africa

**Keywords:** Sierra Leone, HIV, 95-95-95, viral load, differentiated service delivery, stigma, supply chain, drug resistance, implementation science

## Abstract

*Background:* Sierra Leone, with a low adult HIV prevalence (~1.6–1.7%), faces uneven progress toward the UNAIDS 95-95-95 targets, with significant gaps in diagnosis, viral load (VL) coverage, and key population reach.

*Methods:* A narrative review (January 2013–June 2024; final search 30 June 2024) synthesized national reports and peer-reviewed articles on Sierra Leone’s HIV cascade and systemic determinants. Data focused on diagnosis, treatment, suppression, and cross-cutting issues (e.g., stigma, supply chain).

*Results:* In 2023, of an estimated 82,000 people living with HIV (PLHIV), 80% (65,600) were diagnosed. Of those diagnosed, 87% (57,072) were on antiretroviral therapy (ART), and 44% of those on ART (25,112) were virally suppressed. VL testing covered 68% of ART patients (38,812), suggesting incomplete coverage may underestimate true population-level suppression if untested patients are less likely to be suppressed. Key bottlenecks include limited VL platforms, commodity stockouts of antiretrovirals, stigma (especially for female sex workers), adolescent retention gaps, absent drug resistance surveillance, fragmented data systems, and donor-dependent financing.

*Conclusions:* Progress is tangible but fragile. Prioritizing detection (first 95) through scaled testing, alongside VL network strengthening, differentiated service delivery (e.g., six-month multi-month dispensing), stigma monitoring, drug resistance surveillance, and sustainable financing roadmaps, is essential. Targeted research on implementation and cost-effectiveness will support equitable attainment of HIV 2030 goals.

## Introduction

Global consensus on the UNAIDS 95-95-95 targets reframes HIV control by emphasizing a cascade where 95% of people living with HIV (PLHIV) know their status, 95% of those diagnosed receive sustained antiretroviral therapy (ART), and 95% of those on treatment achieve viral suppression by 2025, aiming to end AIDS as a public health threat by 2030 [1]. In sub-Saharan Africa (SSA), aggregate progress masks intra-country disparities, necessitating granular analysis of structural barriers to high coverage. Sierra Leone’s adult HIV prevalence (~1.6–1.7%) is low compared to eastern and southern Africa (often > 5%), potentially due to higher male circumcision rates in West Africa, associated with reduced transmission risk, alongside differences in sexual networks and historical epidemic spread [1, 2]. However, health system recovery from past shocks (e.g., Ebola, COVID-19, Mpox) continues to challenge HIV service delivery [1–4].

HIV testing, often referred to as the first 95 of the UNAIDS 95-95-95 targets, remains a critical challenge in Sierra Leone’s efforts toward epidemic control, with only approximately 80% of PLHIV knowing their status as of recent estimates [1]. The third 95 viral suppression among those on ART appears to be the weakest link in the cascade. According to UNAIDS data and country reports up to 2022, viral suppression rates in Sierra Leone are estimated at approximately 44% among all PLHIV (not just those on treatment), reflecting significant gaps in achieving sustained viral load (VL) suppression [2, 3]. The treatment coverage (second 95) stands at approximately 87% of those diagnosed, indicating a moderate gap compared to the other cascade elements [1, 2]. National strategic planning integrates HIV within broader health platforms, yet rural access barriers, human resource constraints, commodity antiretroviral stockouts, and stigma disrupt equilibrium across the cascade [3–6]. While national data show upward trends, misalignment with the Joint United Nations Programme on HIV/AIDS (UNAIDS) definitions (e.g., VL coverage vs. suppression) creates interpretive ambiguity, which this review clarifies [2–5].

This narrative synthesis is timely as the programmatic focus shifts from service expansion to optimization of quality, retention, and equity. It coalesces disparate data to reveal structural weaknesses threatening sustainability, maps financing for donor transition pathways, and considers emergent technologies (e.g., point-of-care VL assays) for equitable introduction [7, 8]. Objectives are to: (1) synthesize progress toward 95-95-95 benchmarks; (2) identify systemic enablers and barriers (laboratory, supply chain, stigma, financing); (3) highlight research gaps (drug resistance, differentiated service delivery); and (4) propose strategic priorities for 2030 targets. This review’s novelty lies in disaggregating cascade accuracy challenges and linking systems determinants to time-sequenced policy actions in a low-prevalence, resource-constrained setting transitioning to optimization.

## Materials and Methods

### Review rationale and objectives

This narrative review summarizes Sierra Leone’s progress toward UNAIDS 95-95-95 indicators using standard definitions, analyzes structural/programmatic bottlenecks, identifies research and implementation gaps, and proposes prioritized policy actions.

### Conceptual framework

A cascade-oriented framework positioned inputs (governance, financing, commodities, workforce, data systems) as determinants of outputs (testing, ART initiation, retention, VL coverage, suppression) and outcomes (reduced transmission/mortality). Stigma and rights contexts were cross-cutting modifiers [1, 3–11].

### Information sources

Data were sourced from peer-reviewed literature (PubMed, Google Scholar), National AIDS Secretariat (NAS) strategic reports (2013–2024), PEPFAR (President’s Emergency Plan for AIDS Relief) Country Operational Plan summaries, Global Fund performance reports, UNAIDS updates, WHO guidelines, UNICEF briefs, and World Bank indicators. The search period spanned January 2013 to 30 June 2024 (final search). 2023 cascade indicators (e.g., PLHIV estimates, diagnosis, ART coverage, VL suppression) were derived from UNAIDS global updates and PEPFAR summaries, which aggregate data from Sierra Leone’s NAS, Ministry of Health (MoH) surveillance, and spectrum modelling for prevalence/incidence estimates. These secondary sources compile primary programme and facility data, with methodologies detailed in respective reports [1, 5].

### Eligibility and synthesis

Inclusion focused on documents with quantitative or qualitative data on Sierra Leone’s HIV cascade or systemic determinants, and regionally relevant (West/Central Africa) evidence from 2013 to 2024. Exclusions were non-English sources, purely clinical trials, conference presentations, and editorials without empirical content. Extracted variables included PLHIV estimates, diagnosis/treatment/suppression rates, VL coverage, stockouts, stigma, drug resistance surveillance, and financing shares. Narrative synthesis grouped determinants, distinguishing observed data from modelled estimates. Potential biases (e.g., model uncertainty, incomplete VL ascertainment, stigma under-reporting) were considered, with extrapolations from regional evidence explicitly framed [10–21]. Detailed search strategies and data extraction matrices are available in a supplementary file to maintain brevity.

## Results

### Epidemiological overview

As of 2023, Sierra Leone had an estimated 82,000 PLHIV, with prevalence stable at 1.5–1.7% per 2019 Demographic and Health Surveys (DHS), though more recent UNAIDS estimates are prioritized for cascade analysis [1–3]. Of these, 80% (65,600) knew their status, 87% of diagnosed (57,072) were on ART, and 44% of those on ART (47,945) achieved viral suppression (<1000 copies/mL). VL testing covered 68% of ART patients (38,812), with 88% of tested (34,154) conditionally suppressed, suggesting underestimation of population-level suppression if untested patients have poorer outcomes [1–3]. Table 1 provides detailed UNAIDS 95-95-95 cascade data for Sierra Leone in 2023.

**Table 1:** UNAIDS 95-95-95 cascade (standard définitions), Sierra Leone, 2023.

INDICATOR	NUMERATOR	DENOMINATOR	% (95% CI)
Estimated PLHIV	82,000	—	—
Diagnosed (First 95)	65,600	82,000	80%
On ART (Second 95)	57,072	65,600	87%
Virally suppressed (Third 95)	25,112	57,072	44%
VL coverage (≥1 VL test)	38,812	57,072	68%
Conditional suppression (among VL-tested)	34,154	38,812	88%

Notes: Standard UNAIDS definitions applied; Suppression threshold <1000 copies/mL; Conditional suppression may overestimate overall suppression if untested patients are more likely to be unsuppressed. Data sources: UNAIDS 2023; PEPFAR COP 2023. Data derivation: UNAIDS 2023 and PEPFAR COP 2023 figures are based on NAS/MoHS programme data and Spectrum estimates, reflecting national surveillance and facility-level reporting. Interpretation caveat: Improving VL coverage may initially reduce the apparent third 95 as previously untested unsuppressed patients are captured; policymakers should anticipate this artefact.

Key populations (e.g., female sex workers, men who have sex with men) face elevated rates (12–15%), while adolescent girls and vertical transmission contribute to paediatric cases due to gaps in the elimination of mother-to-child transmission programme, including early infant diagnosis [2, 3, 12, 13]. Modelled incidence shows a gradual decline, but slow prevention scale-up (e.g., pre-exposure prophylaxis (PrEP), condom promotion) limits faster progress [3]. Compared to West African peers (e.g., Nigeria ~1.3%, Côte d’Ivoire ~2.4%), Sierra Leone’s burden is modest, yet 95-95-95 progress lags, especially in diagnosis and VL coverage [1–3, 8].

**Figure 1:**
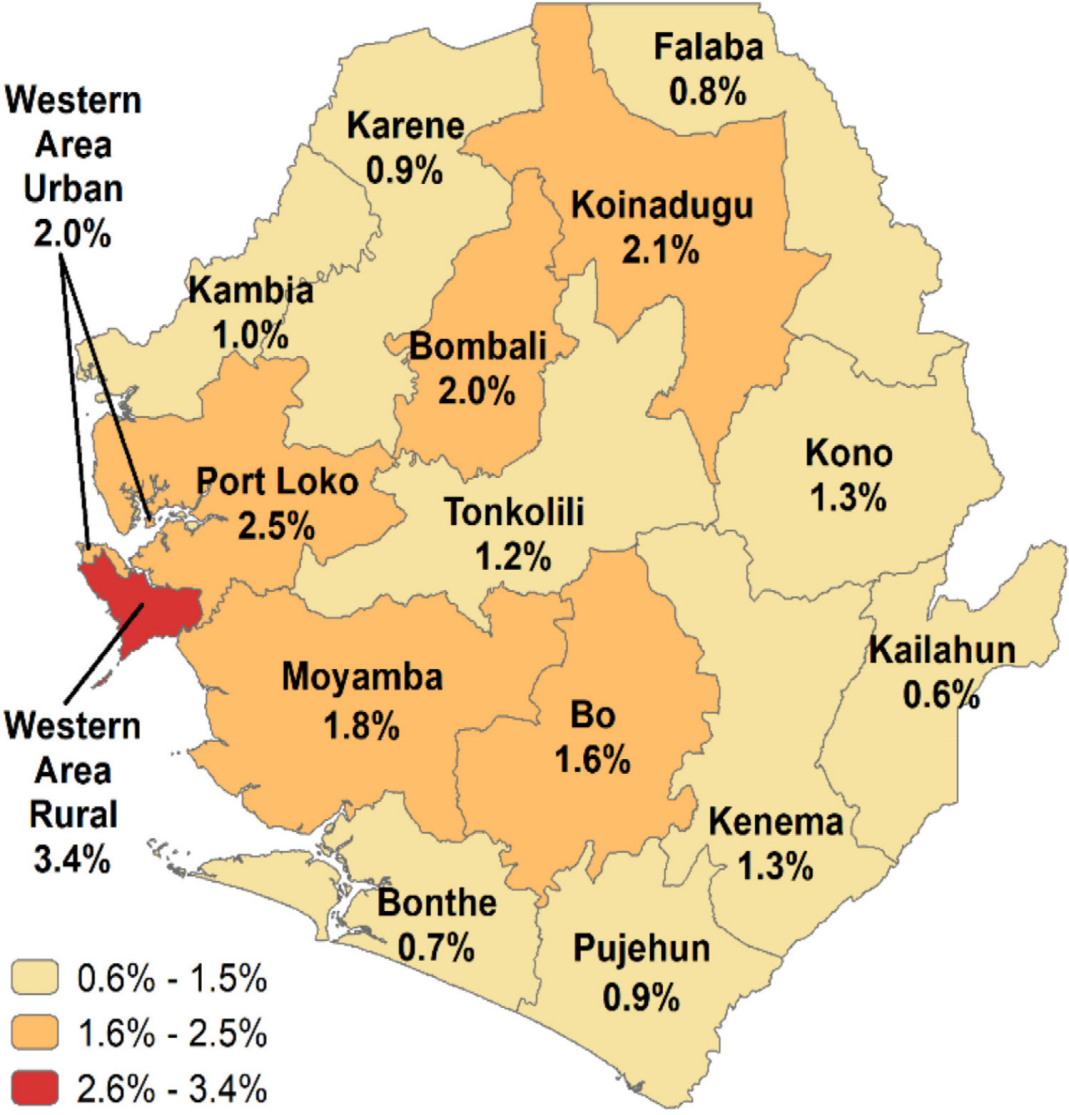
Sierra Leone HIV prevalence by districts (Source: DHS 2019).

Figure 1 illustrates HIV prevalence and 95-95-95 progress by district alongside key VL testing facilities (e.g., Western Area Urban, Bo, Kenema, Makeni, Koidu), highlighting regional disparities [2, 3]. Data granularity is limited for some districts [1–3].

The GeneXpert platform is a rapid, automated molecular diagnostic system that utilizes polymerase chain reaction (PCR) technology to detect and monitor diseases, including HIV. It is designed for near-point-of-care testing, offering quick turnaround times for VL monitoring, which is critical for managing HIV treatment and ensuring viral suppression. The cobas® 5800 System, developed by Roche Diagnostics, is an advanced automated molecular diagnostic instrument specifically engineered for both quantitative and qualitative nucleic acid testing. It provides high-throughput capabilities for VL testing, enabling precise measurement of HIV RNA levels in patient samples, which is essential for assessing treatment efficacy and disease progression. In the context of the Table 2, the presence of these platforms at various hospitals across Sierra Leone indicates the capacity for VL testing, with only Makeni Government Hospital and PCMH/ODCH equipped with both GeneXpert and cobas® 5800 systems, while most facilities rely solely on the GeneXpert platform. Regional disparities persist, with Western regions showing a higher prevalence than Northern/Eastern areas.

**Table 2:** Distribution of viral load testing capacity across districts in Sierra Leone by diagnostic platform.

NO.	HOSPITAL	GeneXpert PLATFORM	C5800 PLATFORM
1	Makeni Government Hospital	x	X
2	Princess Christian Maternity Hospital/Ola During Children’s Hospital	x	X
3	Bo Government Hospital	x	
4	Kenema Government Hospital	x	
5	Connaught Hospital	x	
6	Koidu Government Hospital	x	
7	Portloko Government Hospital	x	
8	34 Military Hospital	x	
9	Kabala Government Hospital	x	
10	Moyamba Government Hospital	x	
11	Kabala Government Hospital	x	
12	Pujehun Government Hospital	x	
13	Kambia Government Hospital	x	
14	Kailahun Government Hospital	x	
15	Magburaka Government Hospital	x	
16	Kamakwie Wesleyan Hospital	x	

### Progress and gaps across 95-95-95

**First 95 (Diagnosis):** Nationally, 80% of PLHIV (65,600/82,000) are diagnosed, the lowest indicator and critical for epidemic control. Disparities exist by sex (women 92%, men 85%) and age (15–24: 77%, 50+: 94%), with young males least aware. Barriers include stigma, test kit stockouts, and rural access gaps; opportunities lie in self-testing and community outreach [1–3, 22]. Provider-initiated testing in antenatal and TB clinics nears saturation (e.g., > 98% TB patients tested), but index testing and male engagement lag [3–5, 8]. (See Supplementary Table 1 for detailed progress toward 95-95-95 targets by sex and age group). (See Supplementary Table 2 for principal barriers and opportunities across the 95-95-95 cascade).

**Second 95 (Treatment):** Of those diagnosed, 87% (57,072/65,600) are on ART, with women (86%) and older adults (89%) outperforming adolescents and young people (15–24 years) (70%). ART scale-up, aligned with test-and-treat and dolutegravir rollout, reduces pre-ART attrition, yet retention is challenged by transport costs, disclosure issues, and stockouts. Differentiated service delivery (DSD), including three-month dispensing (all districts) and pilot six-month multi-month dispensing (MMD), improves adherence, though rural scale-up is limited [1, 3, 15, 23]. Peer support aids retention but faces stipend discontinuity [14–16].

**Third 95 (Suppression):** Of those on ART, 44% (25,112/57,072) are suppressed, though only 68% received a VL test, with 88% of those tested suppressed. This discrepancy suggests underestimation if untested patients fare worse. VL platforms (six total) are urban-concentrated, with delays in sample transport and result turnaround. Adolescents and young people (46% suppression among tested) and rural areas lag, necessitating near point-of-care solutions [1–3, 8, 24].

### Key populations and equity

Key populations face heightened risks. Female sex workers (prevalence ~11%) encounter inconsistent condom use and economic pressures, with peer-led testing increasing uptake, but retention is challenged by mobility and harassment [11, 14, 25]. Men who have sex with men, lacking robust data, avoid facilities due to layered stigma, needing discreet self-testing and safe clinics [11, 14]. Adolescents and young people struggle with confidentiality and transition to adult care, showing lower diagnosis (77%) and suppression (46%), requiring adolescents and young people to be responsive to DSD [12, 13, 25]. Pregnant women benefit from antenatal testing but face postpartum retention gaps, while orphans and vulnerable children risk adherence lapses due to caregiver instability [3, 12, 26]. Data on mobile/mining workers is sparse, warranting targeted outreach [3, 4]. (See Supplementary Table 3 for current data availability and priority interventions for key populations.)

### Key systemic barriers

Systemic challenges constrain Sierra Leone’s HIV response. Stigma, spanning anticipated and enacted forms, deters service uptake, especially among key populations, with limited monitoring or grievance mechanisms [11, 14, 26]. Workforce shortages and uneven urban–rural distribution limit service quality, despite task-shifting to nurses and other cadres of healthcare workers [27]. Fragmented data systems without universal patient identifiers hinder cascade tracking and cohort analysis [28, 29]. Supply chain gaps, including forecasting inaccuracies and stockouts, disrupt commodity availability, exacerbated by poor real-time visibility [30]. Donor-dependent financing (e.g., PEPFAR, Global Fund) risks sustainability amid fiscal constraints, with potential post-2024 drawdowns beyond this review’s scope (data up to June 2024); a domestic funding roadmap is urgent [31, 32]. Past shocks (Ebola, COVID-19, Mpox) exposed resilience gaps, though MMD mitigated some disruptions [32]. Addressing these requires integrated logistics systems, stigma reduction tools, digital interoperability, and sustainable financing strategies [34–35].

### Strategic policy implications

**Service delivery:** Shift from expansion to optimization by prioritizing diagnosis (first 95) through scaled self-testing via pharmacies, academic institutions, and community networks, supported by peer navigators [22, 28]. Universalize six-month MMD for stable adults, decongesting clinics, and integrate adolescent-responsive hours to narrow retention gaps [15, 32].

**Laboratory and supply chain:** Optimize VL access with hub-and-spoke networks and near point-of-care devices for priority groups (e.g., pregnant women, adolescents, and young people), supported by barcode tracking and maintenance contracts [8, 24, 33]. Deploy integrated logistics systems for stock visibility to prevent stockouts [30].

**Stigma and rights:** Institutionalize stigma reduction via facility sensitizations, confidential grievance systems, and community dialogues with faith leaders, monitoring impact with validated scales [14, 25].

**Financing and data:** Develop a medium-term domestic financing roadmap to mitigate donor transition risks, leveraging DSD efficiencies [31]. Harmonize unique patient identifiers and interoperable electronic records for accurate cascade tracking [26, 29]. (See Supplementary Table 5 for detailed systems bottlenecks, priority actions, indicators, and timelines.)

### Limitations of this narrative review

This review synthesizes heterogeneous sources with variability in indicator definitions and data quality, limiting comparability. Reliance on 2019 DHS for some prevalence data may not reflect current trends, though 2023 UNAIDS/PEPFAR figures are prioritized [1–3]. Non-systematic search methods may omit grey literature, and national aggregates obscure district-level disparities. Sparse data on key populations, adolescent and young people adherence, and post-June 2024 events (e.g., 2025 donor funding changes) restrict analysis; future research should address these gaps. Absence of real-time drug resistance data limits assessment of dolutegravir resistance patterns [16, 18]. (See Supplementary Table 4 for prioritized research and implementation evidence gaps, including drug resistance surveillance and cost-effectiveness studies.)

## Conclusion

Sierra Leone has advanced along the 95-95-95 ladder, yet progress remains uneven, with VL suppression (44%) as the weakest link, critical for epidemic control, alongside gaps in VL monitoring (68% coverage) and adolescent and young people and key population retention. Consolidating gains hinges on optimization: closing the diagnosis gap with scaled testing strategies, strengthening VL networks for faster turnaround, universalizing six-month MMD, embedding rights-based stigma reduction, adolescents and young people, care prioritized and integrating digital tools for data integrity. Accurate cascade reporting, robust VL and drug resistance surveillance, and sustainable financing roadmaps are pivotal to leveraging the country’s modest epidemic size. Transparent annual scorecards tracking cascade accuracy, VL coverage, MMD scale, stigma indicators, and domestic funding shares will ensure accountability toward 2030 goals.

## Data Availability

Data sharing is not applicable as no primary datasets were generated or analyzed for this study. However, for access to the extracted data or further information on the sources reviewed, please contact the corresponding author, Dr. Ibrahim Franklyn Kamara, at ikamara@who.int or +232 76 345757.

## Patient and Public Involvement

Patients and/or the public were not involved in the design, conduct, reporting, or dissemination plans of this research.

## Additional Files

The additional files for this article can be found as follows:

Supplementary Table 1. Progress towards the UNAIDS 95-95-95 targets in Sierra Leone.

Supplementary Table 2. Principal barriers and opportunities across the 95-95-95 HIV cascade in Sierra Leone.

Supplementary Table 3. Key populations: current data and gaps.

Supplementary Table 4. Prioritised research and implementation evidence gaps.

Supplementary Table 5. Systems bottlenecks, priority actions, indicators, timeline.

This finding of multiple local introgression events has at least three key implications.

## References

[r1] Joint United Nations Programme on HIV/AIDS (UNAIDS). Global AIDS Update 2023. Geneva: UNAIDS; 2023.

[r2] Joint United Nations Programme on HIV/AIDS (UNAIDS). Sierra Leone Country Factsheet 2023. Geneva: UNAIDS; 2023.

[r3] National AIDS Secretariat (NAS) Sierra Leone. National HIV Strategic Plan 2021–2025. Freetown: NAS; 2021.

[r4] Ministry of Health and Sanitation, Sierra Leone. Health Sector Recovery Plan 2015–2020. Freetown: MoHS; 2015.

[r5] United States President’s Emergency Plan for AIDS Relief (PEPFAR). Sierra Leone Country Operational Plan 2023 Strategic Direction Summary. Washington, DC: PEPFAR; 2023.

[r6] The Global Fund. Grant Performance Report HIV Sierra Leone 2023. Geneva: The Global Fund; 2023.

[r7] World Health Organization. Consolidated Guidelines on HIV Testing Services 2019 Update. Geneva: WHO; 2019.

[r8] World Health Organization. Consolidated Guidelines on the Use of Antiretroviral Drugs for Treating and Preventing HIV Infection: Recommendations for a Public Health Approach 2021 Update. Geneva: WHO; 2021.

[r9] World Bank. World Development Indicators: Sierra Leone Population Total 2023. Washington, DC: World Bank; 2023.

[r10] World Health Organization. Updated Recommendations on HIV Viral Load and Early Infant Diagnosis Testing 2021. Geneva: WHO; 2021.

[r11] Joint United Nations Programme on HIV/AIDS (UNAIDS). Confronting Discrimination: Overcoming HIV-Related Stigma and Discrimination in Health-Care Settings and Beyond. Geneva: UNAIDS; 2020.

[r12] United Nations Children’s Fund (UNICEF). Women and Children and HIV in West and Central Africa 2023 Report. New York: UNICEF; 2023.

[r13] United Nations Children’s Fund (UNICEF). Paediatric HIV Gap Report 2023. New York: UNICEF; 2023.

[r14] Joint United Nations Programme on HIV/AIDS (UNAIDS). Key Populations in West and Central Africa: Evidence Brief 2022. Geneva: UNAIDS; 2022.

[r15] World Health Organization. Policy Brief: Update on Dolutegravir Use for HIV Treatment in All Populations 2019. Geneva: WHO; 2019.

[r16] World Health Organization. HIV Drug Resistance Report 2022. Geneva: WHO; 2022.

[r17] International AIDS Society. Differentiated Service Delivery for HIV Treatment: A Decision Framework 2021 Update. Geneva: IAS; 2021.

[r18] World Health Organization. Consolidated Guidelines on HIV Prevention, Testing, Treatment, Service Delivery and Monitoring: Recommendations for a Public Health Approach 2023. Geneva: WHO; 2023. 34370423

[r19] Joint United Nations Programme on HIV/AIDS (UNAIDS). Long Acting Antiretrovirals and the Response to HIV: Considerations for Equitable Introduction 2023. Geneva: UNAIDS; 2023.

[r20] World Health Organization. Ebola Situations Report June 2016. Geneva: WHO; 2016.

[r21] Elston JWT, Cartwright C, Ndumbi P, Wright J. The health impact of the 2014–2015 Ebola cutbreak. Public Health. 2017;143:60–70. 28159028 10.1016/j.puhe.2016.10.020

[r22] World Health Organization. Guidelines on HIV Self-Testing and Partner Notification: Supplement to Consolidated Guidelines on HIV Testing Services. Geneva: WHO; 2016. 27977094

[r23] International AIDS Society. The Differentiated Service Delivery Evidence Reviews 2022. Geneva: IAS; 2022.

[r24] Unitaid. HIV Diagnostics Technology Landscape 2022. Geneva: Unitaid; 2022.

[r25] Joint United Nations Programme on HIV/AIDS (UNAIDS). Global Partnership for Action to Eliminate All Forms of HIV-Related Stigma and Discrimination 2021 Progress Report. Geneva: UNAIDS; 2021.

[r26] World Health Organization. Consolidated Guideline on Sexual and Reproductive Health and Rights of Women Living with HIV 2017. Geneva: WHO; 2017. 28737847

[r27] World Health Organization. Task Shifting Global Recommendations and Guidelines. Geneva: WHO; 2008.

[r28] World Health Organization. WHO Guideline: Recommendations on Digital Interventions for Health System Strengthening 2019. Geneva: WHO; 2019. 31162915

[r29] World Health Organization. Smart Guidelines: Digital Adaptation Kits for HIV 2022. Geneva: WHO; 2022.

[r30] USAID | DELIVER PROJECT. Final Country Report: Strengthening HIV Commodity Security in Sierra Leone 2017. Arlington, VA: USAID | DELIVER; 2017.

[r31] Joint United Nations Programme on HIV/AIDS (UNAIDS). Financing the HIV Response: UNAIDS HIV Financial Resources 2022. Geneva: UNAIDS; 2022.

[r32] Joint United Nations Programme on HIV/AIDS (UNAIDS). In Danger: Global AIDS Update 2022. Geneva: UNAIDS; 2022.

[r33] Clinton Health Access Initiative. Strengthening Viral Load and Early Infant Diagnosis Sample Transport Systems 2021 Report. Boston, MA: CHAI; 2021.

[r34] World Health Organization. Partner Notification for HIV: Technical Brief 2019. Geneva: WHO; 2019.

[r35] United Nations Children’s Fund (UNICEF). Improving Early Infant Diagnosis Coverage and Turnaround Times in West and Central Africa 2022 Briefing. New York: UNICEF; 2022.

