# Supplementary material for: Advancing Toward the UNAIDS 95-95-95 Targets in Sierra Leone: A Narrative Review of Progress, Persistent Gaps, and Policy Priorities
Source: Ann Glob Health. 2026 Mar 26;92(1):27. doi: 10.5334/aogh.5152 (PMC13025156; doi:10.5334/aogh.5152)
Supplement: Supplementary Table 1. — Progress towards the UNAIDS 95-95-95 targets in Sierra Leone. [file agh-92-1-5152-s1.pdf]

Table 1: Progress towards the UNAIDS 95-95-95 targets in Sierra Leone

Indicator	National Estimate (%)	Disaggregated by Sex	Disaggregated by Age Group	Comments
Proportion of PLHIV who know their status	89%	Male: 85%<br>Female: 92%	15-24: 77%<br>25-49: 91%<br>50+: 94%	Lower awareness in young males
Proportion of diagnosed PLHIV on ART	83%	Male: 78%<br>Female: 86%	15-24: 70%<br>25-49: 85%<br>50+: 89%	Gaps in adolescent linkage
Proportion of PLHIV on ART who received a viral load test	65%	Male: 62%<br>Female: 68%	15-24: 55%<br>25-49: 67%<br>50+: 70%	Rural areas lag behind
Proportion of those tested with viral suppression (<1000 c/ml)	59%	Male: 54%<br>Female: 63%	15-24: 46%<br>25-49: 61%<br>50+: 65%	Adolescents are at the highest risk

*****PLHIV = People Living with HIV; ART = Antiretroviral Therapy
