# Supplementary material for: Advancing Toward the UNAIDS 95-95-95 Targets in Sierra Leone: A Narrative Review of Progress, Persistent Gaps, and Policy Priorities
Source: Ann Glob Health. 2026 Mar 26;92(1):27. doi: 10.5334/aogh.5152 (PMC13025156; doi:10.5334/aogh.5152)
Supplement: Supplementary Table 2. — Principal barriers and opportunities across the 95-95-95 HIV cascade in Sierra Leone. [file agh-92-1-5152-s2.pdf]

Table 2: Principal barriers and opportunities across the 95-95-95 HIV cascade in Sierra Leone

95 Domain	Principal Barriers	Corresponding Opportunities/Interventions	Rationale & Expected Impact Pathway
First 95: Testing & Diagnosis	- Test kit stockouts<br>- Stigma & fear<br>- Low rural coverage	- Supply chain visibility tools<br>- Community-led testing<br>- Self-testing kits	- Improved logistics ensure kit availability<br>- Community approaches reach hidden populations
Second 95: ART Coverage & Adherence	- ART stockouts<br>- Poor retention/adherence<br>- Long travel distance	- Stock monitoring dashboards<br>- Peer support/adherence clubs<br>- Community ART groups	- Reduces loss to follow-up<br>- Brings services closer to clients
Third 95: Viral Suppression	- Limited lab capacity<br>- Delayed viral load turnaround<br>- Inconsistent monitoring	- Sample tracking systems<br>- Near point-of-care viral load machines<br>- Task-shifting for sample collection	- Faster results enable timely clinical action<br>- Decentralized testing improves coverage
