# Supplementary material for: Advancing Toward the UNAIDS 95-95-95 Targets in Sierra Leone: A Narrative Review of Progress, Persistent Gaps, and Policy Priorities
Source: Ann Glob Health. 2026 Mar 26;92(1):27. doi: 10.5334/aogh.5152 (PMC13025156; doi:10.5334/aogh.5152)
Supplement: Supplementary Table 3. — Key populations: current data and gaps. [file agh-92-1-5152-s3.pdf]

Table 3: Key populations: current data and gaps

Population	Current Data Availability	Estimated Prevalence (if any)	Surveillance Gap	Priority Interventions Needed
Female Sex Workers	Limited ad hoc outreach data	No published estimate[1]	No recent IBBS	Peer-led testing, PrEP readiness, flexible hours
Men Who Have Sex with Men	Sparse, mostly anecdotal	No published estimate[1]	No IBBS; safety concerns	Discreet self-testing, stigma-safe clinics, violence reporting
Mobile/Mining Workers	Minimal	—	Occupational risk unquantified	Workplace testing, tailored MMD
Transport Workers	Minimal	—	No behavioural data	Corridor outreach + self-testing

* IBBS-integrated biological and behavioural surveillance; MMD- Multi-months dispensing
