# Supplementary material for: Advancing Toward the UNAIDS 95-95-95 Targets in Sierra Leone: A Narrative Review of Progress, Persistent Gaps, and Policy Priorities
Source: Ann Glob Health. 2026 Mar 26;92(1):27. doi: 10.5334/aogh.5152 (PMC13025156; doi:10.5334/aogh.5152)
Supplement: Supplementary Table 4. — Prioritised research and implementation evidence gaps. [file agh-92-1-5152-s4.pdf]

Table 4: Prioritised research and implementation evidence gaps

Gap	Priority (High/Med)	Proposed Design	Lead / Partners	Decision Use	Timeline
Sentinel HIV drug resistance surveillance (integrase + NNRTI)	High	Sentinel cohort genotyping (WHO early warning indicators)	NAS Lab Directorate / Partners	Guide regimen policy & early switch criteria	Initiate ≤12 mo
Cost-effectiveness of DSD models (facility vs. MMD vs. community ART groups)	High	Pragmatic comparative implementation + micro-costing	MoHS + Econ Research Unit	Resource allocation for scale	12–24 mo
Stigma reduction intervention effectiveness	High	Stepped wedge cluster trial (facility/community)	NAS + CSOs	Scale validated package	18–36 mo
Rural VL access optimisation (hub-spoke vs near POC)	Medium	Operational modelling + pilot	Lab Directorate + CHAI	Investment planning	12–24 mo
Adolescent adherence & retention determinants	High	Mixed-method cohort + qualitative	Adolescent Health Unit	Tailored adolescent DSD	12–24 mo
Digital interoperability feasibility (EMR–LIS–UPI)	Medium	Implementation research	eHealth Unit	National digital roadmap	12–18 mo
Economic evaluation of stigma & peer navigator models	Medium	Cost–utility & cost per suppressed patient	Health Econ partners	Budget advocacy	18–30 mo
