# Supplementary material for: Advancing Toward the UNAIDS 95-95-95 Targets in Sierra Leone: A Narrative Review of Progress, Persistent Gaps, and Policy Priorities
Source: Ann Glob Health. 2026 Mar 26;92(1):27. doi: 10.5334/aogh.5152 (PMC13025156; doi:10.5334/aogh.5152)
Supplement: Supplementary Table 5. — Systems bottlenecks, priority actions, indicators, timeline. [file agh-92-1-5152-s5.pdf]

**Table 5: Systems bottlenecks, priority actions, indicators, timeline**

| Domain | Bottleneck | Priority Action | Indicator (Baseline → Target) | Timeline | Lead |
|---|---|---|---|---|---|
| Cascade Accuracy | Misaligned definitions | Publish standardised national cascade with numerators | Official cascade bulletin released | ≤6 mo | NAS |
| Viral Load | Limited coverage & long turnaround | Implement VL optimisation plan (routing + tracking) | VL coverage [%] (X→Y); median TAT days (X→≤14) | 12–24 mo | Lab Directorate |
| DSD | Limited 6-month MMD | Scale 6-month MMD for stable adults | % stable on 6M MMD (X→≥70%) | 12 mo | MoHS Pharm |
| Drug Resistance | No surveillance | Launch sentinel genotyping | Sites enrolled (0→≥5); annual DR report | ≤18 mo | Lab Directorate |
| Stigma | No routine monitoring | Implement facility stigma monitoring tool | Facilities with stigma baseline (0→≥50%) | 12 mo | NAS + CSOs |
| Supply Chain | Visibility gaps | Deploy integrated LMIS with near-real-time reporting | Facilities reporting stock data (X→≥90%) | 18 mo | CMS |
| Data Systems | Fragmentation & no UPI | Pilot UPI + EMR–LIS integration | Sites with functional UPI (0→≥20 high-volume) | 12–18 mo | eHealth Unit |

| | | | | | |
|---|---|---|---|---|---|
| Adolescents | Retention gap | Youth-friendly DSD + digital adherence pilot | 12m retention (X→+10 pp) | 24 mo | Adolescent Unit |
| Financing | Donor dependence | Sustainability roadmap with budget targets | Domestic share commodities (X→X+Δ%) | 24 mo | MoHS + Finance |
| Peer Support | High turnover | Standardise cadre roles & stipends | Sites with formal peer role (X→≥80%) | 18 mo | NAS + HR Unit |
